# A Multifaceted Approach to Treatment of Recalcitrant Cutaneous Periorbital Juvenile Xanthogranuloma

**DOI:** 10.18502/jovr.v18i1.12733

**Published:** 2023-02-21

**Authors:** Alexandra Van Brummen, Sarah Jacobs, Shu Feng, Emily Li, Arash J. Amadi

**Affiliations:** ^1^Department of Ophthalmology, University of Washington, Seattle, WA, USA; ^2^Department of Ophthalmology, University of Alabama, Birmingham, AL, USA; ^3^Wilmer Eye Institute, Johns Hopkins University, Baltimore, MD, USA

**Keywords:** CO2 Laser, Juvenile Xanthogranuloma, Laser-assisted Steroid Delivery

## Abstract

**Purpose:**

To demonstrate novel treatments for patients with high juvenile xanthogranuloma (JXG) eyelid lesion burden.

**Case Report:**

A 14-year-old girl was referred to the oculoplastic surgery service for management of worsening extensive bilateral eyelid and adnexal lesions in the setting of JXG. The patient underwent intra-lesional steroid injections, serial excisions, and reconstruction with skin grafts. She was subsequently treated with CO ${}_{2}$ laser-assisted topical steroid application, which resulted in lesion regression.

**Conclusion:**

A novel multimodal approach to treatment of severe periocular JXG, incorporating surgical debulking, skin autograft, CO2 laser, and intra-lesional steroids, can be effective for lesion control.

##  INTRODUCTION 

Juvenile xanthogranuloma (JXG) represents one of the most common manifestations of non-Langerhans cell histiocytosis.^[1]^ Patients typically present with red–yellow papules or nodules, which occur more extensively in the pediatric population compared to adult patients.^[1,2]^ JXG lesions most commonly present cutaneously, however, they can affect intraocular structures in 0.3–10% of pediatric cases.^[3]^ In rare cases, JXG has also been reported to involve pulmonary, pericardial, testicular, hepatic, central nervous system, and skeletal system tissues.^[4]^ Management varies depending on the location involved and the extent of disease burden, spanning a range from observation to topical steroid application to surgical excision with or without radiation treatment.^[2,3]^ The authors present a rare case of JXG with extensive periorbital soft tissue involvement that required multi-modal management including surgical excision, skin grafting, and laser-assisted drug delivery of intra-lesional steroids. Collection and evaluation of protected patient health information was in compliance with the Health Insurance Portability and Accountability Act of 1996 and adhered to the ethical principles outlined in the Declaration of Helsinki as amended in 2013. Informed consent for use of photos was obtained from the patient.

##  CASE REPORT 

**Figure 1:**
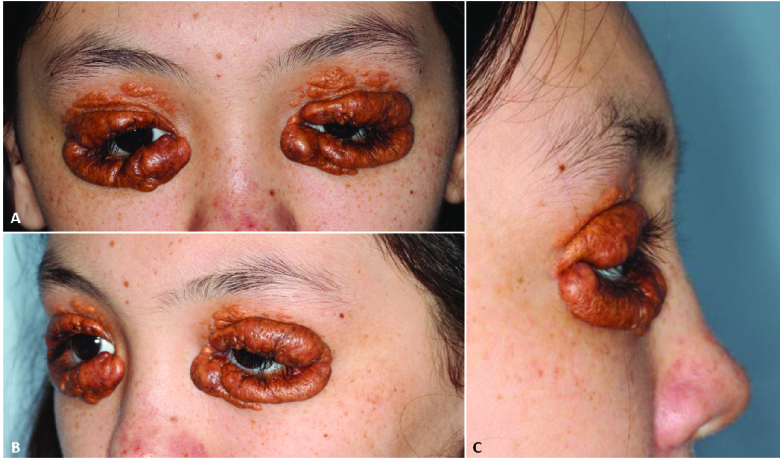
Demonstration of extensive upper and lower lid lesions (A, B, C) with satellite lesions extending towards upper brow.

**Figure 2:**
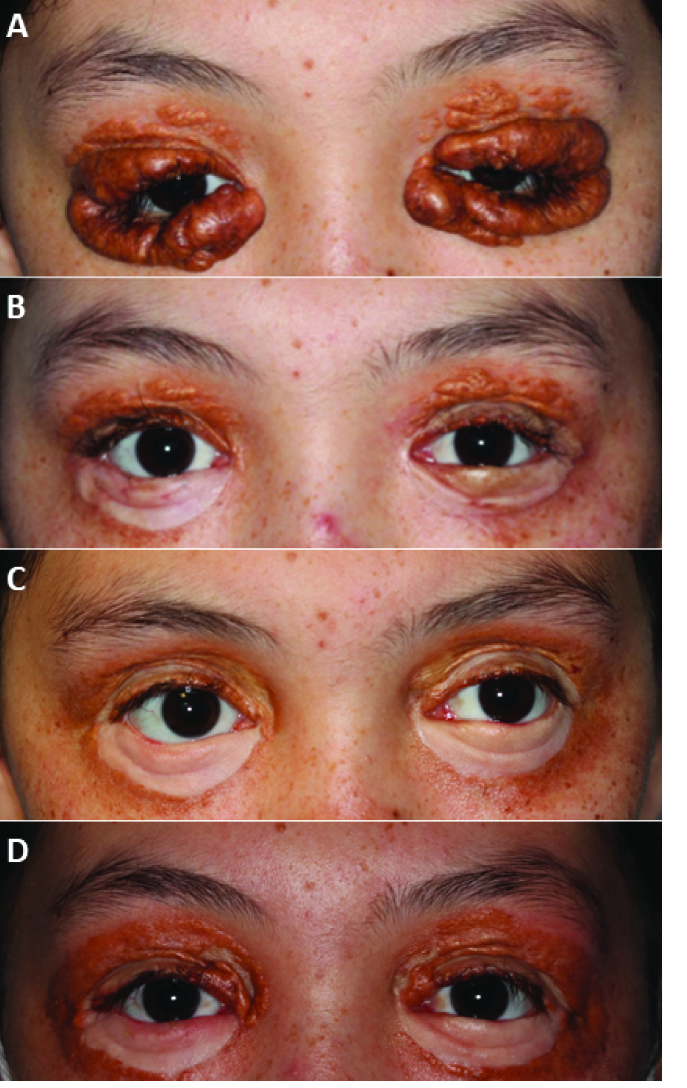
Periorbital lesions preoperatively (A) compared to status post-surgical excision and intralesional steroid application (B). (C) Postoperative month three after CO ${}_{2}$ laser resurfacing. (D) Demonstration of persistent regression of lesions 39 months postoperatively, with only mild recurrence.

A 14-year-old female presented to ophthalmology clinic as a referral for JXG involving the bilateral eyelids and sinus passages. The skin lesions were first noted around age eight and increased in size until age ten. She had previously undergone surgical debulking for extensive JXG infiltration of the sinonasal mucosa. On examination, her Snellen visual acuity was 20/20 bilaterally. Intraocular pressure and extraocular motility were normal. She had numerous protuberant lesions on the upper and lower eyelids bilaterally [Figures 1 & 2A]. Full dilated examination ruled out ocular involvement.

Per patient preference, the periocular skin lesions were observed for a year after presentation to ophthalmology department. When no spontaneous lesion regression occurred during that time, the patient agreed to proceed with serial lesion excision with skin grafting, one eyelid at a time, each separated by a two-week interval. Full-thickness skin autografts were obtained from retro-auricular skin for the bilateral lower eyelids and upper inner arm skin for the upper eyelids. Intralesional injections of triamcinolone acetonide (40 mg/mL) were given in the upper eyelids at two-week intervals prior to lesion excision (at four and two weeks preoperatively in the right upper lid; and six, four, and two weeks preoperatively in the left upper lid). Silicone scar gel was utilized postoperatively to help with scar modulation of the skin grafts on all four eyelids, starting postoperative week three and continuing through postoperative month three. The patient healed well without excessive trichiasis or other recurrence of lesions [Figure 2B]. At postoperative month three, the patient had persistent lesions along the outer perimeter of her skin graft sites. She underwent serial treatments of CO ${}_{2}$ laser resurfacing (four total treatments, given at two-to-three-month intervals), utilizing the Lumens UltraPulse CO ${}_{2}$ laser (Lumenis, Yokne'am Illit, Israel). For each treatment, the SCAAR-Fx setting (5% density, 80 mJ, 200 Hz) was applied to the preseptal region and DeepFx setting (10% density, 15 mJ, 250 Hz) was applied to the entire periocular zone. Immediately after the laser application, topical triamcinolone acetonide (40 mg/mL) was massaged into the treated skin, which allows for laser-assisted drug delivery.^[5]^ The patient demonstrated further improvement after CO ${}_{2}$ laser resurfacing [Figure 2C].

At the most recent follow-up 39 months after lesion excision, examination revealed lesion regrowth on the superonasal upper lids [Figure 2D]. The involved areas were small, and the patient was not bothered by them functionally or cosmetically. Her exam otherwise demonstrated well-healed skin grafts and an overall acceptable outcome. Further treatment with intra-lesional steroids was offered, but the patient deferred them in pursuit of naturopathic treatments.

##  DISCUSSION 

JXG is the most common non-Langerhans cell histiocytosis, typically presenting in childhood with red–yellow papules or nodules.^[1,2]^ On histopathology, involved skin cells, derived from dermal dendrites, express CD1a but do not exhibit reactivity for S-100 protein or contain Birbeck granules.^[2]^ The disease can present with systemic involvement, affecting the brain, eye, lungs, liver, spleen, and other organs.^[2]^ Failure to recognize systemic involvement can result in fatal consequences for patients. Freyer et al described a case initially characterized as isolated cutaneous JXG, subsequently found to have intracranial disease when the patient developed seizures one month after initial diagnosis, warranting systemic treatment with chemotherapy.^[2]^ Conditions including neurofibromatosis type I (NF-1) and juvenile myelomonocytic leukemia can additionally present with JXG.^[3]^ In patients younger than three years of age, 18% of those diagnosed with NF-1 were also found to have JXG.^[3,6]^ In addition, patients with NF-1 and JXG have a 20 to 30 times higher risk of developing comorbid juvenile myelomonocytic leukemia.^[3,7]^ Diagnosis of JXG warrants further serologic evaluation with a complete blood count and comprehensive metabolic panel to screen for hepatic, renal, and hematopoietic abnormalities.

Achieving sustained regression of JXG lesions remains a clinical challenge. Surgical excision and chemotherapy with vinblastine, methotrexate, and others play roles in systemic management.^[2]^ For intracranial lesions, the treatment of choice has typically been described as surgical excision, at times supplemented by radiation.^[8]^

Sustained regression of isolated cutaneous lesions also poses a clinical challenge. Some suggest a period of observation as cutaneous lesions have been known to spontaneously regress.^[1]^ For persistent cases, the mainstay of treatment includes intra-lesional or topical corticosteroids.^[3,9]^ Elner et al reviewed six cases demonstrating improvement in symptoms of orbital and eyelid xanthogranuloma, including improvement of lid ptosis and diplopia, with triamcinolone acetonide 40 mg/mL injections alone.^[9]^ Similarly, Kuruvilla et al described regression of an eyelid JXG lesion in an infant in response to intra-lesional steroid injection.^[10]^ However, neither reported complete resolution of clinical evidence of disease; rather, they described partial regression, which alleviated disease symptoms. In addition, the lesion burden of the described patients was mild compared to the case presented in our report.

For more severe cases with larger or multiple lesions, treatment modalities extend beyond corticosteroids. One author described elimination of multiple JXG lesions without recurrence after CO ${}_{2}$ laser treatment.^[11]^ Others have also described successful use of CO ${}_{2}$ laser in treatment of necrobiotic xanthogranuloma lesions in the setting of lymphoplasmacytic lymphoma.^[12]^ Finally, surgical excision has been described in some cases of cutaneous lesions as well, especially in cases of large disfiguring or debilitating lesions in young children.^[3]^

In summary, the authors present a multifaceted approach to treatment of cutaneous periorbital JXG recalcitrant to local corticosteroid treatment alone. A combination of intra-lesional steroid injection, surgical lesion debulking, skin graft reconstruction, and CO ${}_{2}$ laser skin resurfacing with laser-assisted drug delivery produced great improvement in the patient's functional and cosmetic concerns, maintained for several years before partial relapse. Because a multipronged approach to treatment was used, it is not possible to discern with certainty that the laser-assisted steroid delivery was the main reason for improvement, as the steroid injections may also have contributed to the results. Future investigation includes optimization of CO ${}_{2}$ laser treatment regimen (total number of treatments, treatment interval, and subsequent maintenance treatments for localized lesion recurrence) and steroid delivery.

##  Declaration of patient consent 

The patient agreed to the publication of this case report and photos, and a signed University of Washington media release for publication was completed.      

##  Financial Support and Sponsorship 

EL is the endowed James L. Hargiss, M.D., Ophthalmic Plastic and Reconstructive Surgery Fellow and the Rayment Endowed Fellow in Ophthalmology. The department is funded by an unrestricted grant from Research to Prevent Blindness.

##  Conflicts of interest

The authors have no financial or conflicts of interest disclosures.
